# RSSM-Based Virtual Sensing and Sensorless Closed-Loop Control for a Multi-Temperature-Zone Continuous Crystallizer

**DOI:** 10.3390/s26051698

**Published:** 2026-03-07

**Authors:** Mingrong Dong, Hang Liu, Geng Yang, Lin Lu, Jia’nan Zhao

**Affiliations:** 1 Faculty of Information Engineering and Automation, Kunming University of Science and Technology, Kunming 650500, China; liuhang0707@stu.kust.edu.cn (H.L.); yanggeng@stu.kust.edu.cn (G.Y.); lulin@stu.kust.edu.cn (L.L.); zhaojianan@stu.kust.edu.cn (J.Z.); 2 Yunnan Key Laboratory of Computer Technologies Application, Kunming University of Science and Technology, Kunming 650500, China

**Keywords:** electric continuous crystallizer, virtual sensor, RSSM, world model, reinforcement learning

## Abstract

Precise temperature control is crucial for maintaining product quality and optimizing energy efficiency in multi-zone continuous crystallizers. However, such industrial processes typically exhibit complex nonlinear dynamics and strong coupling effects. More critically, physical constraints often prevent sensor installation, rendering temperatures in key regions unobservable and challenging traditional closed-loop control strategies. To address partial observability and model uncertainty, this paper proposes a Model-Based Reinforcement Learning (MBRL) framework utilizing solely offline historical data. The core innovation lies in developing a Recursive State Space Model (RSSM) that serves not only as a high-fidelity digital twin but, more critically, is deployed as a real-time “virtual sensor” to infer unobservable system states. This virtual sensing capability provides precise state estimates for downstream policy optimization. Additionally, a multi-objective reward function is designed to balance tracking error, stability, and control cost. Experimental results demonstrate that the proposed virtual sensor exhibits exceptional long-term stability, maintaining high fidelity and effectively suppressing error accumulation during long-term multi-step autoregressive predictions. Consequently, the trained agent outperforms traditional Proportional-Integral-Derivative (PID) and Model Predictive Control (MPC) controllers, achieving over 67% improvement in temperature tracking accuracy while reducing control action costs by more than 93%, indicating smoother system operation and enhanced energy efficiency.

## 1. Introduction

High-precision process control serves as the cornerstone of modern energy- and material-intensive industries, directly impacting product quality, production efficiency, and energy consumption. In metallurgy, the accuracy and stability of heat-treatment processes are crucial to ensuring product performance and economic viability. As the core equipment in tin pyrometallurgical refining, the electrically heated continuous crystallizer is of significant importance for tin-lead separation and purification due to its innovative spiral-conveying structure and countercurrent mass transfer mechanism. In China, this technology has been extensively integrated into tin purification processes. Its operation relies on the tin-lead (Sn-Pb) binary alloy phase diagram, achieving separation by establishing a precise temperature gradient ranging from 32 °C to 183 °C within the crystallization tank.

However, achieving autonomous optimization control for this system presents significant challenges. It is a typical multiple-input multiple-output (MIMO) system [1,2,3], characterized by strong thermal conduction and material-flow coupling among its five temperature-controlled zones. More critically, the process involves complex phase-change nonlinear dynamics and significant time delays. In practical industrial scenarios, controlling such complex processes primarily confronts three core challenges. First, regarding model uncertainty [4,5,6], the complex physicochemical mechanisms make it extremely difficult to establish precise mechanistic mathematical models. Second, the high cost of data and interaction [7,8,9] presents a significant barrier; the excessive risks and high data interaction costs associated with online trial-and-error learning make secure data collection extremely challenging. Third, and most critically, is the issue of partial state observability [10,11]. Due to the unique helical structure and harsh operating conditions—such as high temperatures and multiphase flow—traditional invasive temperature sensors cannot be deployed in critical liquid-phase regions. This prevents real-time acquisition of precise state information in key temperature zones, severely hindering the effective implementation of advanced closed-loop control strategies.

To overcome reliance on precise mechanistic models, reinforcement learning (RL) offers a highly promising technical pathway [12,13,14]. However, mainstream RL paradigms exhibit limitations when addressing the aforementioned challenges. Model-free online RL requires substantial interaction samples, and its exploration process may involve unsafe actions, making it challenging to meet industrial safety requirements [15,16]. While offline RL can leverage historical data, it faces severe out-of-distribution (OOD) generalization challenges [17,18,19] and cannot fundamentally resolve partial observability issues caused by missing sensors [20,21].

To systematically address the aforementioned challenges, this paper proposes a Model-Based Reinforcement Learning (MBRL) framework [22]. Its core idea is to first learn a dynamic model of the environment (i.e., the “world model”) [23] from limited offline data, and then utilize it as a high-fidelity digital twin [24,25] to safely and efficiently optimize control strategies [26]. Specifically, we adopt the Recurrent State Space Model (RSSM) as the core architecture [27]. RSSM combines the memory capability of deterministic states for long-term dependencies (e.g., significant time delays) with the modeling capability of stochastic states for uncertainties (e.g., phase-transition dynamics), making it highly suitable for the complex characteristics of crystallization processes. Despite the emergence of novel sequence models like S5 [28] and Mamba [29], RSSM remains a reliable choice for addressing such industrial challenges due to its mature theoretical foundation and robustness in modeling nonlinear dynamics. This paper aims to bridge the gap between advanced generative sequence models and complex industrial process control. Our experimental results demonstrate that the constructed RSSM serves not only as an environment simulator for policy optimization but also as a high-precision real-time “virtual sensor.” Experimental data reveal that the model exhibits exceptional robustness in long-term multi-step autoregressive predictions, accurately reconstructing system dynamic trajectories without significant drift. Based on this high-fidelity virtual sensor, the trained agent achieves closed-loop control without physical sensor feedback. Compared to traditional Proportional Integral Derivative (PID) and Model Predictive Control (MPC), this approach improves temperature control accuracy by over 67% while reducing energy costs associated with control actions by more than 93%, significantly enhancing system stability and energy efficiency.

To systematically address the aforementioned challenges and achieve these results, this study’s specific contributions are summarized as follows. First, an industrial-grade digital twin and virtual sensor based on the RSSM were constructed. By leveraging historical offline data, a high-fidelity world model of the crystallization process was successfully built. Its ability to accurately reconstruct system dynamics by inferring hidden states in the absence of physical sensor feedback was validated through experiments, effectively addressing partial-observability challenges. Second, a multi-objective reward function was designed to address diverse industrial control objectives. This function incorporates temperature tracking, process stability, and control costs. Using Bayesian optimization [30,31,32], the optimal weight configuration was determined, ensuring the control strategy strikes a balance between precision and energy efficiency. Finally, the feasibility of sensorless closed-loop control was validated. Experimental results demonstrate that the deployed world model can function as a real-time virtual sensor to drive closed-loop control solely through control feedback, thereby establishing a new paradigm for intelligent control in sensor-constrained environments.

## 2. Problem Modeling

This study aims to design an advanced autonomous control strategy for a multi-stage electro-thermal continuous crystallizer. The core challenges of this system lie in its complex, nonlinear, multivariable, and coupled dynamics, as well as the “partial observability” issue arising from the inability to directly measure temperatures in critical zones. To address these challenges, a Model-Based Reinforcement Learning (MBRL) framework is constructed. This framework formalizes the control problem as a Partially Observable Markov Decision Process (POMDP) [33]. Subsequently, a Recurrent State-Space Model (RSSM) is learned as a “world model” from offline data to capture latent dynamics, thereby effectively overcoming the partial-observability challenge. Finally, within an Actor-Critic framework [34], the control policy is optimized through “imagination” in the learned latent space. The following sections provide a detailed exposition of the proposed method from multiple perspectives: theoretical formalization, model construction, training mechanisms, policy optimization, and objective function design.

The electro-thermal continuous crystallizer is a typical multiple-input multiple-output (MIMO) system. Heat conduction and material flow between its five temperature-controlled sections exhibit significant coupling. Simultaneously, the thermal characteristics during heating and cooling exhibit high nonlinearity, and control actions suffer from non-negligible time delays (dead time).

Since the system’s critical states (internal temperatures) cannot be directly observed via physical sensors, this control problem is no longer a standard Markov Decision Process (MDP) but a Partially Observable Markov Decision Process (POMDP). POMDP provides a robust mathematical framework for sequential decision-making in uncertain environments. The control problem for the crystallizer is formalized as a septuple:

(1) $$M=\left(S,A,T,R,\Omega ,O,\gamma \right)$$

(1) State Space S: A set comprising the system’s physical states. At any given time step t, the state $s_{t}\in S$ is a vector describing the system’s conditions in the crystallizer. It primarily consists of the temperatures of the five temperature-controlled sections, including internal temperatures of the crystallizer that are not directly measured by sensors. This actual physical state is not directly observable. It is estimated and represented by the world model’s latent state.
(2) Action Space A: Given the structural characteristics and multi-zone thermal dynamics of the crystallizer described above, the control action space is designed to manage these localized thermal requirements effectively. Specifically, the controller actions are defined as a discrete set of operations. Four operations are possible for each of the five temperature-controlled sections: heating only, cooling with water spray only, taking no action, and simultaneous heating and water spray. Consequently, the total action space is the Cartesian product of the individual action sets for these five sections, resulting in a composite action space with $4^{5}=1024$ possible actions. At each time step t, the specific action executed by the agent is denoted as $a_{t}\in A$.
(3) State Transition Function T: $T\left(s_{t+1}|s_{t},a_{t}\right)$ The state transition function describes the probability of transitioning to the next state $s_{t+1}$, from the current state $s_{t}$ after executing action $a_{t}$. The complex interplay of principles from heat transfer, fluid dynamics, and crystallization kinetics governs these system behaviors. This function is unknown and is the core target that the world model needs to learn from data.
(4) Reward Function R: $R\left(s_{t},a_{t}\right)$, scalar function designed to guide the agent toward achieving the multiple control objectives of precise temperature tracking, process stability, and energy efficiency. Based on the current state and the action taken, the immediate reward of that decision is evaluated.
(5) Observation Space Ω: The set of all information the agent can directly measure from the environment at any given time step. In the context of this study, the composition of the observation vector $o_{t}$ distinguishes between the offline training and online deployment phases. Specifically, during the offline training phase, $o_{t}$ encompasses historical records of actual temperatures across all zones, allowing the world model to accurately learn the system dynamics via supervised learning. Conversely, during the online deployment or sensorless control phase, observation is strictly constrained by physical limitations, rendering real-time temperature readings inaccessible to the agent. Under these conditions, the observation $o_{t}$ is limited to control feedback, such as the operational status of heaters and valves. It is effectively uninformative about the system’s internal thermodynamic state. This severe partial observability necessitates that the agent infer the system’s latent state solely from the action history using the learned world model.
(6) Observation Function O: $O\left(o_{t+1}|s_{t+1},a_{t}\right)$, This function defines the probability of receiving an observation $o_{t+1}$ given the new state $s_{t+1}$ and the preceding action $a_{t}$. It describes the relationship between true internal states and external sensor readings, which may involve delays and noise. Similar to the state transition function, this function is also unknown and is learned implicitly by the world model.
(7) Discount Factor γ: $\gamma \in \left[0,1\right)$, This parameter balances the trade-off between immediate and future rewards, ensuring that the cumulative reward over an infinite horizon is a finite value. For industrial control tasks aimed at long-term stable operation, a value close to 1 is typically chosen.

The core challenge of a POMDP is that the agent cannot directly access the actual state $s_{t}$ and must base its decisions on a history of observations and actions. This reliance on history necessitates memory-based decision-making approaches, such as using a Recurrent Neural Network (RNN) to construct a state representation.

Since the actual state is unknown, a rational agent cannot act based on a single, speculative state. Instead, it must make decisions based on a belief state $b\left(s\right)$. The belief state is a probability distribution over the entire space of true state S, which $b_{t}\left(s_{t}\right)$ denotes the probability that the system is in state s at the time step t. This belief state is a sufficient statistic for the entire history $\left(a_{0},o_{1},a_{1},o_{2},\ldots ,a_{t-1},o_{t}\right)$ of action-observation, meaning it encapsulates all historical information required for optimal decision-making. Given the previous belief state $b_{t}$ the executed action $a_{t}$ and the new observation $o_{t+1}$ the new belief state $b_{t+1}\left(s_{t+1}\right)$ can be updated via Bayesian filtering:

(2) $$b_{t+1}\left(s_{t+1}\right)=\eta \cdot O\left(o_{t+1}|s_{t+1},a_{t}\right)\sum_{s_{t}\in S}T\left(s_{t+1}|s_{t},a_{t}\right)b_{t}\left(s_{t}\right)$$

where η is a normalization constant that ensures $b_{t+1}$ is a valid probability distribution (i.e., the sum of its probabilities over all states is 1), this update process comprises two steps: prediction and update.

First, a predicted belief, $\hat{b_{t+1}}\left(s_{t+1}\right)$, is computed based on the previous belief and the transition model:

(3) $$\hat{b_{t+1}}\left(s_{t+1}\right)=\sum_{s_{t}\in S}T\left(s_{t+1}|s_{t},a_{t}\right)b_{t}\left(s_{t}\right)$$

Next, this predicted belief is updated with the new observation using the observation model:

(4) $$b_{t+1}\left(s_{t+1}\right)=\eta \cdot O\left(o_{t+1}|s_{t+1},a_{t}\right)\hat{b_{t+1}}\left(s_{t+1}\right)$$

It is worth noting that, in the sensorless control scenario, the real-time observation of the critical temperatures is unavailable due to physical constraints. Consequently, the posterior belief update (Equation (4)) cannot be explicitly computed using sensor feedback. Instead, the system operates in an open-loop belief prediction mode based on Equation (3). The validity of this approach relies on the high fidelity of the learned transition function T (parameterized by the World Model), which acts as a ‘virtual sensor’ to propagate the belief state solely based on the action history $a_{t}$.

Despite this operational constraint, a POMDP can, in theory, be reformulated as a fully observable MDP over a continuous belief space, known as a “Belief MDP.” However, this transformation is often impractical in real-world applications. Since the belief space (i.e., the space of all possible state distributions) is continuous and high-dimensional, the number of belief states becomes infinite even when the original state space is finite. This inherent complexity is the fundamental motivation for employing functional approximators such as the Recurrent State-Space Model (RSSM).

To solve a POMDP problem, an agent must implicitly or explicitly accomplish two core tasks: (1) state estimation (updating its belief about the world state) and (2) control (selecting optimal actions based on that belief). The Model-Based Reinforcement Learning (MBRL) framework suits these two tasks well.

The MBRL approach first focuses on learning a model of the world’s dynamics (i.e., transition and observation functions). This learned model can then be used to perform state estimation (similar to the filtering process in Kalman filters [35] or hidden Markov models) and to plan or learn a control policy using the estimated states. In contrast, model-free methods often struggle in POMDPs. They typically attempt to directly learn a mapping from history to action without constructing an explicit intermediate state representation. While theoretically feasible, this end-to-end approach often suffers from extremely low sample efficiency in practice due to the need to handle extremely high-dimensional input spaces (history sequences).

## 3. Methodology

**Figure 1:**
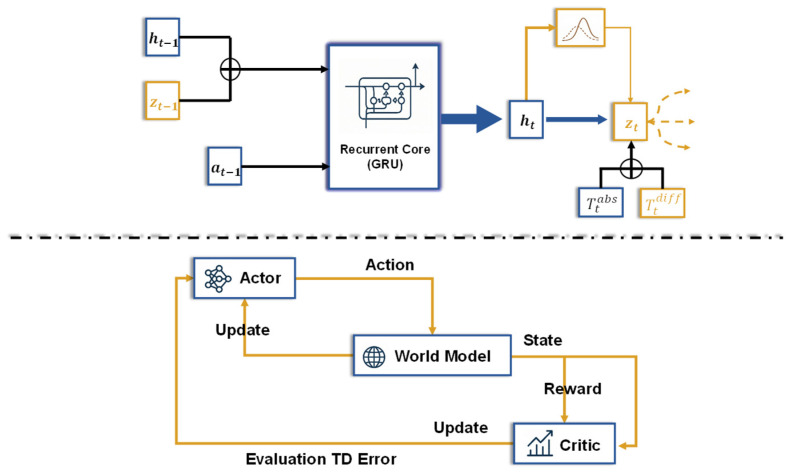
Diagram of the Overall Agent Architecture. This architecture consists of two components. (1) A Recurrent State-Space Model (RSSM, **upper part**), which learns a world model by encoding observations into a latent state composed of a deterministic state $h_{t}$ and a stochastic state $z_{t}$ through auto-encoding and dynamic prediction. (2) An Actor-Critic module (**lower part**), which efficiently trains a policy network (Actor) and a value network (Critic) by imagining trajectories generated within the learned world model to optimize the agent’s behavior.

This section primarily describes the model’s specific implementation methods, and the overall agent architecture is illustrated in Figure 1. To achieve the optimal control of a crystallizer using a Model-Based Reinforcement Learning (MBRL) approach, a Recurrent State-Space Model (RSSM) is first employed as the core architecture. The structure of the RSSM, which is composed of a mixture of deterministic and stochastic components, enables it to robustly learn long-term historical information through the deterministic path while capturing inherent process noise and model uncertainty via the stochastic path. Subsequently, an Actor-Critic reinforcement learning method interacts with the RSSM temporal model to learn the optimal control policy.

### 3.1. RSSM World Model

The RSSM primarily comprises a deterministic state model that stores historical temperature and operational data. This component consists of multiple Gated Recurrent Unit (GRU) layers.

(5) $$h_{t}=f_{0}\left(h_{t-1},z_{t-1},a_{t-1}\right)$$

Following this is the stochastic state model component. The stochastic state is sampled from a probability distribution parameterized by a neural network. This module learns a compressed, abstract representation of the underlying system state. Two critical probability distributions are then defined. First, the prior distribution predicts the state based solely on the deterministic history:

(6) $$p_{\theta }\left(z_{t}|h_{t}\right)=N\left(\mu_{\theta }\left(h_{t}\right),\sigma_{\theta }\left(h_{t}\right)\right)$$

where N denotes the multivariate Gaussian distribution, and $\mu_{\theta }$ and $\sigma_{\theta }$ represent the mean and standard deviation vectors, respectively, which are predicted by the neural network based on the deterministic state $h_{t}$.

Next is the posterior representation model, which refines state estimation by incorporating the actual absolute temperature and temperature differential observations (acquired directly from the system’s sensors) at the current time step. This posterior distribution is defined as:

(7) $$q_{\theta }\left(z_{t}|h_{t},T_{t}^{abs},T_{t}^{diff}\right)=N\left(\mu_{\theta }\left(h_{t},T_{t}^{abs},T_{t}^{diff}\right),\;\sigma_{\theta }\left(h_{t},T_{t}^{abs},T_{t}^{diff}\right)\right)$$

where $\mu_{\theta }$ and $\sigma_{\theta }$ represent the parameters of the posterior distribution inferred from both history and current observations. The representation model primarily corrects the transition model’s state estimation but is not utilized during actual model inference. The hidden state distribution at the current time step is generated directly using the transition model.

The RSSM framework characterizes the system dynamics by decoupling the state representation into a deterministic hidden state ($h_{t}$) and a stochastic latent state ($z_{t}$)—specifically, the deterministic hidden state $h_{t}$ functions as the system’s explicit memory. Updated via a multi-layer Gated Recurrent Unit (GRU) network (Equation (5)), $h_{t}$ robustly memorizes and propagates long-term historical dependencies, such as the crystallizer’s significant thermal delays and sequential operational inputs. Conversely, the stochastic latent state $z_{t}$ is designed to model inherent unobservable uncertainties, such as process noise and complex phase-transition dynamics, which cannot be perfectly predicted by deterministic history alone. $z_{t}$ is formulated as a multivariate Gaussian distribution. During offline training, the posterior representation model fuses $h_{t}$ with current and differential temperature observations to generate an accurate posterior distribution of $z_{t}$ (Equation (7)). Concurrently, the transition model predicts the prior distribution of $z_{t}$ relying solely on $h_{t}$ (Equation (6)). Finally, to reconstruct the physical observations, the deterministic state $h_{t}$ and the stochastic state $z_{t}$ are concatenated into a unified feature vector ($h_{t}⊕z_{t}$). This combined representation is then processed by a multi-layer perception (MLP) decoder to predict the observation parameters, allowing the model to leverage both long-term macroscopic trends and short-term stochastic variations.

The observation and reward model uses the learned hidden state to predict physically meaningful observations. Specifically, the decoder employs a multi-head architecture, where each of the five temperature zones is indicated by an independent output head to capture localized dynamics and avoid feature interference. The five temperature prediction heads serve as the model’s primary outputs, reconstructing the physical state vector from the latent state and acting as a “soft sensor”.

(8) $$p_{\theta }\left(T_{t}^{\mathrm{abs}}|h_{t},z_{t}\right)=N\left(\mu_{\theta }\left(h_{t},z_{t}\right),\sum_{\theta }\left(h_{t},z_{t}\right)\right)$$

Additionally, a separate reward prediction head predicts immediate rewards, a component crucial for efficiently training the reinforcement learning agent entirely within the learned world model.

(9) $$p_{\theta }\left(r_{t}|h_{t},z_{t}\right)=N\left(\mu_{\theta }\left(h_{t},z_{t}\right),1\right)$$

The goal of training the world model is to maximize the Evidence Lower Bound (ELBO) on the log-likelihood of observational trajectories in an offline dataset *D*. Its objective function is given by:

(10) $$L\left(\theta \right)=\sum_{t=1}^{T}E_{q_{\theta }\left(z_{t-1},a_{t-1},T_{t}^{abs}\right)}\left[logp_{\theta }\left(T_{t}^{\mathrm{abs}},r_{t}|z_{t}\right)\right]\;-\beta \cdot D_{\mathrm{KL}}\left(q_{\theta }\left(z_{t}|z_{t-1},a_{t-1},T_{t}^{\mathrm{abs}},T_{t}^{\mathrm{diff}}\right)∥p_{\theta }\left(z_{t}|z_{t-1},a_{t-1}\right)\right)$$

This objective function consists of two key components. The first is a reconstruction term that compels the model to accurately reconstruct the actual sequences of temperatures and rewards from the learned latent states. The second is a regularization term in the form of a Kullback–Leibler (KL)-divergence penalty between the prior, $p_{\theta }\left(z_{t}|h_{t}\right)$, and the posterior, $q_{\theta }\left(z_{t}|h_{t},T_{t}^{\mathrm{abs}},T_{t}^{\mathrm{diff}}\right)$. Here, the coefficient β serves as a regularization hyperparameter that controls the trade-off between the observation reconstruction accuracy and the information capacity of the stochastic latent state. This penalty makes the transition model, $p_{\theta }\left(z_{t}|h_{t}\right)$, learn to perform accurate single-step predictions without access to the current observations $\left(T_{t}^{\mathrm{abs}},T_{t}^{\mathrm{diff}}\right)$.

Crucially, a distinction is made between training and deployment. During offline training, the posterior estimator $q_{\theta }$ (Equation (7)) is used to correct state estimates using available temperature data. However, for sensorless control, the posterior estimator is deactivated since real-time observations $o_{t}$ are unavailable. Instead, the system relies strictly on the prior transition model $p_{\theta }\left(z_{t}|h_{t}\right)$ (Equation (6)) to infer the stochastic latent state. This enables the model to function as a self-contained ‘virtual sensor,’ propagating the system state solely based on historical actions.

The open-loop prediction capability of the Recurrent State-Space Model (RSSM) is fundamental to this research. Once the world model is trained, it can predict the entire future trajectory of the system based solely on an initial state and a sequence of future control actions. In practical industrial applications, the mechanical constraints of the crystallizer render traditional intrusive and non-intrusive temperature measurement methods infeasible. The RSSM can serve as a rapid simulator to infer the crystallizer’s core temperature in real time. The prediction process is initialized by inferring an initial hidden state, $h_{0},\;z_{0}$, from historical non-intrusive control feedback data. For all subsequent time steps (*t* > 0), the model predicts the temperature evolution solely based on measurable control feedback.

### 3.2. Actor-Critic (AC) Model

Furthermore, a high-performance control strategy is learned using an Actor-Critic reinforcement learning algorithm, which leverages the RSSM-based temperature inference simulator. This Actor-Critic framework consists of two neural networks—an actor and a critic—trained collaboratively.

Actor (Policy Network),$\;\pi_{\theta }\left(a_{t}|s_{t}\right)$: This network is responsible for learning the control policy. It maps a given state $s_{t}$, which is composed of the latent state variables $\left(h_{t},z_{t}\right)$, to a probability distribution over actions. The objective is to adjust the policy parameters θ to maximize the expected cumulative future reward (the expected return).

Critic (Value Network), $V_{\psi }\left(s_{t}\right)$: This network estimates the state-value function, representing the expected cumulative future reward from a given state $s_{t}$. It aims to provide the Actor with a low-variance learning signal to guide policy updates.

The Critic network updates its value estimates using the λ-return mechanism. This approach constructs a weighted average of single-step Temporal Difference (TD) estimates and multi-step returns, with the weights determined by a decay coefficient λ. By bootstrapping from the value of subsequent states across different horizons, this mechanism effectively balances the bias-variance trade-off, thereby enhancing the Critic network’s generalization. Specifically, the state value function estimate is defined as:

(11) $$V_{\lambda }\left(s_{t}\right)=\left(1-\lambda \right)\sum_{n=1}^{H-1}\lambda^{n-1}G_{t}^{\left(n\right)}+\lambda^{H-1}G_{t}^{\left(H\right)}$$

where H denotes the look-ahead prediction horizon (the maximum number of steps for bootstrapping), and the index n represents the specific number of look-ahead steps for each return estimate. Here, $G_{t}^{\left(n\right)}$ represents the *n*-step return, the sum of discounted rewards accumulated over *n* steps from time step t, plus the discounted value of the resulting state at step t+n. It is formally defined as:

(12) $$G_{t}^{\left(n\right)}=\sum_{k=0}^{n-1}\gamma^{k}r_{t+k}+\gamma^{n}V_{\psi }\left(s_{t+n}\right)$$

where $\gamma \in \left[0,1\right]$ is the discount factor that determines the present value of future rewards, and $r_{t+k}$ is the immediate reward at step t+k.

To improve the precision of the value function estimates, the Critic network updates its parameters ψ by minimizing the mean squared error (MSE) between the predicted value $V_{\psi }\left(s_{t}\right)$ and the aforementioned λ−return target $V_{\lambda }\left(s_{t}\right)$. The loss function is therefore defined as:

(13) $$L\left(\psi \right)=E\left[{\left(V_{\lambda }\left(s_{t}\right)-V_{\psi }\left(s_{t}\right)\right)}^{2}\right]$$

The actor network is optimized using the policy gradient method for policy updates. The objective is to adjust the parameters ϕ to increase the likelihood of selecting actions that yield higher-than-average returns. To this end, the gradient of the policy objective, $\nabla_{\phi }J\left(\phi \right)$, is estimated as:

(14) $$\nabla_{\phi }J\left(\phi \right)\approx E\left[\sum_{t=1}^{H}\nabla_{\phi }\log\pi_{\phi }\left(a_{t}|s_{t}\right)\cdot A_{t}\right]$$

Here, the advantage function $A_{t}$ quantifies how much better an action is compared to the average policy performance at the state $s_{t}$ level. It is defined as:

(15) $$A_{t}=V_{\lambda }\left(s_{t}\right)-V_{\psi }\left(s_{t}\right)$$

The advantage function provides an effective baseline that yields a more stable policy gradient and effectively reduces its variance. This collaborative optimization of the Actor and Critic enables the efficient joint learning of both the policy and value functions.

In the equations above, the state $s_{t}$ is the concatenation of the deterministic hidden state, ht, and the stochastic latent state $z_{t}$:

(16) $$s_{t}=h_{t}⊕z_{t}$$

where the symbol ⊕ represents the operation of joining two vectors into a single augmented vector.

Additionally, the reward function R used in this Actor-Critic method incorporates three components: temperature control error, temperature stability, and control action cost. The total reward is formulated as follows:

(17) $$R=-\left(w_{\mathrm{error}}\cdot \underset{i=1..5}{\max}\left|T_{\mathrm{current},i}-T_{\mathrm{target},i}\right|+w_{\mathrm{stability}}\cdot \underset{i=1..5}{\max}\left|T_{\mathrm{current},i}-T_{\mathrm{previous},i}\right|+\;w_{\mathrm{action}}\cdot \frac{1}{5}\sum_{i=1}^{5}C\left(a_{i}\right)\right)$$

where $w_{\mathrm{error}}$, $w_{\mathrm{stability}}$, and $w_{\mathrm{action}}$ are the weighting coefficients used to balance the importance of control precision, system stability, and energy-related action costs, respectively. These coefficients allow the agent to fine-tune its behavior to prioritize specific control objectives. Here, $C\left(a_{i}\right)$ is the action cost function. For the discrete action indices $a_{i}$, they correspond to: Cooling Only (0), Heating Only (1), No Action (2), and Simultaneous Heating & Cooling (3). The cost function is defined as:

(18) $$C\left(a_{i}\right)=\left\{\begin{array}{l} 1.0\;\;if\;a_{i}=0\;(\mathrm{Cooling})\\ 1.5\;\;if\;a_{i}=1\;(\mathrm{Heating})\\ 0.0\;\;if\;a_{i}=2\;(\mathrm{No}\;\mathrm{Action})\\ 2.5\;\;if\;a_{i}=3\;(\mathrm{Simultaneous}) \end{array}\right.$$

The specific values in the action cost function $C\left(a_{i}\right)$ (1.0 for Cooling, 1.5 for Heating, and 2.5 for Simultaneous action) are assigned to reflect the relative energy intensity of each discrete control mode. Since these represent discrete operational states, the absolute magnitudes of these weights are less critical than their ability to reflect the hierarchy of energy consumption. We assigned 1.0 to Cooling and 1.5 to Heating to indicate that heating is more energy-intensive in our system. At the same time, the value of 2.5 for Simultaneous operation corresponds to the combined consumption of both modes. As long as these weights accurately capture the relative impact of each action, they provide a sufficient signal for the agent to learn an energy-efficient policy without requiring the values to represent absolute energy units.

## 4. Results

This section presents a comprehensive empirical evaluation of the Recurrent State-Space Model (RSSM), which serves a dual function in our work. It provides the simulation environment for training the reinforcement learning agent and acts as the digital twin of the electric-thermal continuous crystallizer. This dual role imposes stringent requirements on the model’s generalization capability, predictive accuracy, and long-term autoregressive stability. To this end, the RSSM is analyzed on three fronts. First, its convergence dynamics are examined to assess its fundamental learning capabilities. Second, its generalization performance is tested under various data-splitting schemes, and its sensitivity to the training data is systematically investigated. Finally, long-term stress tests are utilized to probe the cumulative error dynamics during autoregressive generation, a core metric for validating its efficacy as a digital twin. Additionally, ablation studies are conducted to clarify the contribution of each architectural component to the model’s overall performance. Subsequently, the reinforcement learning (RL) temperature control policy is systematically evaluated across multiple dimensions. First, to assess generalization, its performance is examined on both the training and validation sets. Since the agent is trained within a world model learned from real-world trajectories, this model serves as regularization. It is crucial to determine if the agent overfits this learned world. This assessment compares reward convergence curves across the training and validation sets and evaluates the policy’s performance stability across different instances of the world model. Second, the performance of the proposed RL policy is compared to several traditional baseline controllers. The reward function’s design is also analyzed by contrasting penalties based on maximum error (MAX operation) with those based on mean error (MEAN operation) for both temperature control and stability terms. Finally, Bayesian optimization is employed to fine-tune the reward function weights, thereby maximizing control performance. This process also allows us to observe the influence of each penalty term on the final temperature control accuracy.

### 4.1. Dataset Description

This section primarily describes the collected data and the methods used.

**Figure 2:**
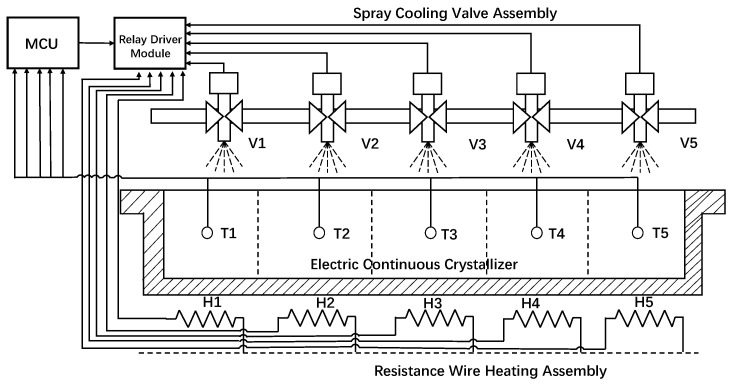
Data Acquisition Platform Framework Diagram.

Figure 2 illustrates the schematic architecture of the data acquisition platform. Positioned beneath the electric continuous crystallizer tank, five independent resistance heating elements (H1–H5) provide thermal energy, while a spray cooling assembly comprising five solenoid valves (V1–V5) is mounted above the tank to facilitate temperature reduction. The system regulates the thermal gradient in the crystallizer by modulating heating and cooling via a central control unit. Concurrently, five custom-designed thermocouples (T1–T5) are embedded within the tank to monitor real-time temperature variations in critical zones. While this configuration enables precise zonal measurement, it introduces hardware complexity and potential stability challenges in industrial environments. The data acquisition process yields five datasets of temperature variations and corresponding control inputs, categorized into four distinct modes: Cooling, Heating, Idle, and Simultaneous. During this continuous data-acquisition process, the sampling interval is strictly set to 5 s, which also defines the duration of a single time step (t). Operationally, within each time step, an activated heating element remains engaged for the full duration, whereas an activated cooling valve opens for only a brief fraction of the interval. To ensure dataset diversity, a PID algorithm is employed to track randomized temperature setpoints within a predefined range, thereby facilitating the exploration of a comprehensive state space. It should be noted that the five thermocouples embedded in the critical zones are part of a specially designed, temporary data acquisition system developed solely for experimental validation. This system utilizes a complex assembly of slip rings, springs, and a customized central rotating shaft to achieve short-term temperature monitoring. While effective for offline data collection and model verification, this apparatus is not suitable for long-term industrial applications due to its high complexity, frequent maintenance requirements, and limited mechanical durability under continuous operational conditions. Therefore, the challenge of partial observability remains a primary constraint in standard industrial settings, necessitating the robust estimation capabilities of the proposed Reinforcement Learning framework.

**Figure 3:**
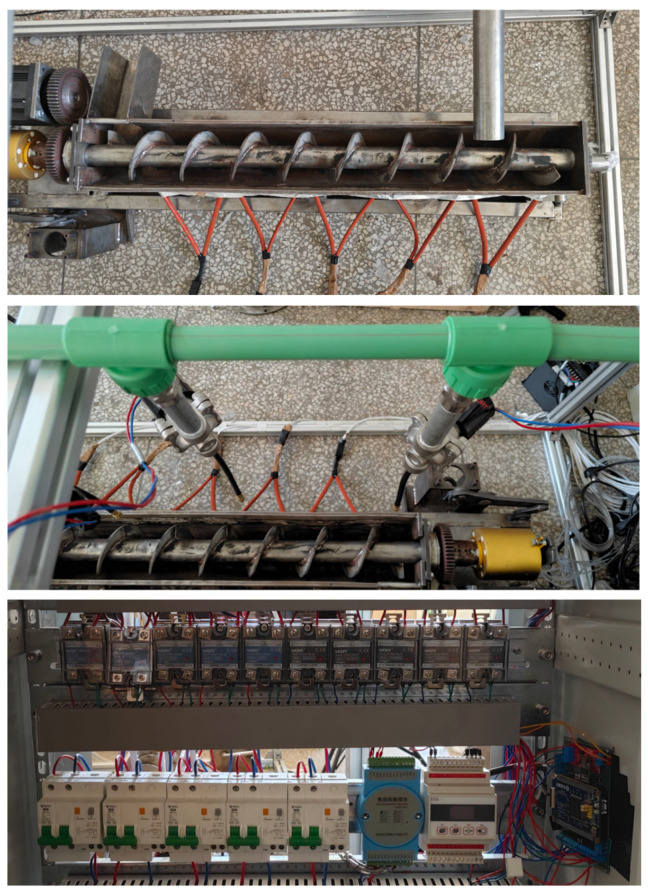
Physical Diagram of the Data Acquisition Platform.

Figure 3 illustrates the physical implementation of the data acquisition platform. The upper panel presents a top-down perspective of the continuous crystallizer structure. The central panel details the spray cooling assembly, which is actuated by solenoid valves. The lower panel displays the electrical control cabinet, anchored by an STM32 microcontroller unit (MCU). Regarding the sensor and actuator placement, the thermocouples and resistance heating elements are strategically integrated within the central rotating shaft and positioned beneath the crystallizer trough, respectively.

**Figure 4:**
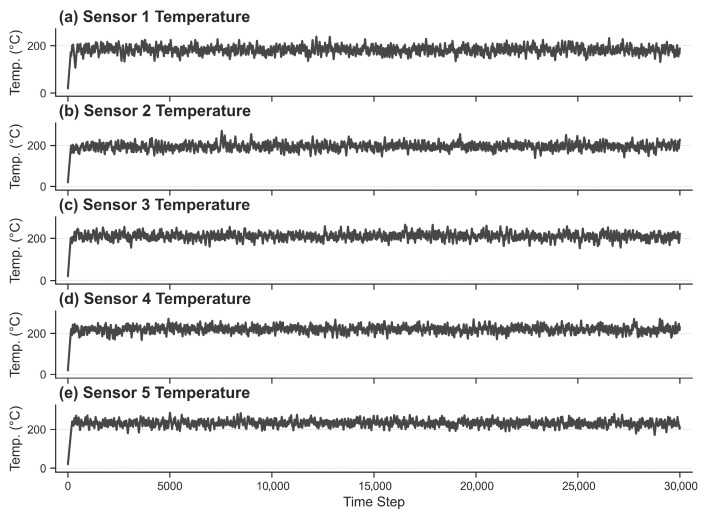
Temperature Change Chart.

Figure 4 illustrates the temporal evolution of the collected temperature data. The dataset encompasses 30,000 discrete time steps, with each interval capturing simultaneous readings from the five distributed sensors.

**Figure 5:**
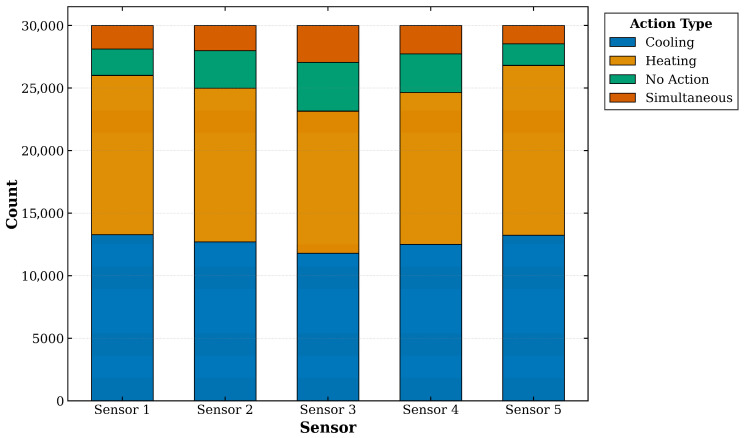
Temperature Control Operating Frequency Chart.

Figure 5 presents the frequency distribution of control actions across the five sensor zones. The dataset comprises 30,000 timesteps, during which the operational states—Cooling, Heating, Idle (No Action), and Simultaneous—were recorded and aggregated for each corresponding crystallizer section.

### 4.2. World Model Validity Experiment

This section will focus on evaluating the Recurrent State Space Model (RSSM), primarily examining its fundamental learning capabilities, generalization performance, and cumulative error. Comparative experiments will be conducted to clarify the impact of each module on the model’s overall performance.

#### 4.2.1. Analysis of RSSM Convergence and Predictive Performance

**Figure 6:**
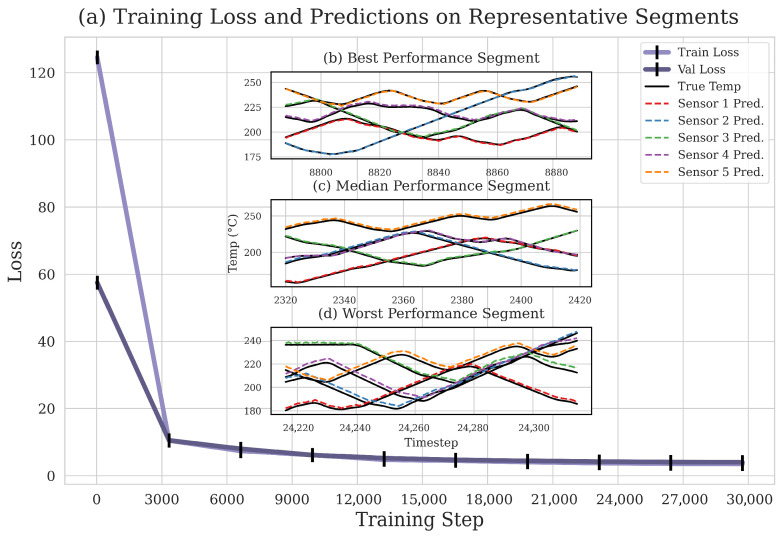
Assessment of the learned dynamics and predictive performance of the RSSM. (**a**) Convergence curves for the training and validation losses. (**b**) Model predictions for the Best Performance Segment, where predicted trajectories, shown as dashed lines, almost perfectly overlap with the ground-truth temperatures, represented by solid lines. (**c**) Predictions for the Median Performance Segment, demonstrating the reproduction of complex and coupled interactions among sensors. (**d**) Predictions for the Worst Performance Segment, which capture the general trends despite more pronounced prediction errors. The X-axes for the three prediction segments display the original time-step indices of these distinct 100-step sequences from the validation set.

The main plot (Figure 6a) displays the training and validation loss curves over 30,000 training steps. To facilitate precise tracking of convergence progress, the X-axis ticks are explicitly marked at 3000-step intervals. The model demonstrates an ideal convergence pattern. The loss drops sharply in the initial phase (approximately the first 3000 steps). This indicates that the model quickly captures the system’s primary dynamics, including the relationships between operational inputs and temperatures, as well as interactions among different temperature zones. After the initial decline, both loss curves enter a plateau and converge to a low, stable value. The minimal gap between the training and validation loss curves suggests that the model generalizes well to unseen data without significant overfitting. This confirms the model has learned a robust and reliable representation of the system’s dynamic characteristics.

To provide a more comprehensive and intuitive assessment of the learned dynamics, the figure includes three inset plots showing the model’s predictive performance on distinct 100-step segments from the validation set. It is essential to clarify that Figure 6b, Figure 6c, and Figure 6d correspond to the Best, Median, and Worst prediction scenarios, respectively. Furthermore, their X-axes display the original time-step indices, indicating the specific locations of these segments within the sequence. In Figure 6b, the model’s predicted trajectories (dashed lines) almost perfectly overlap with the ground-truth temperatures (solid lines), demonstrating the model’s maximum predictive accuracy. Figure 6c, which depicts the model’s median behavior, also shows excellent agreement, reproducing the complex, coupled interactions among sensors, including phase shifts and varying amplitudes. Figure 6d clearly reveals the model’s limitations in the worst-case scenario. While the prediction errors are more pronounced here than in the other segments, the model still captures the general trends and the essence of the cross-fluctuations between sensors. This multi-faceted evaluation demonstrates the model’s high-fidelity predictive capability across various scenarios. The strong performance, even in the most challenging segments, underscores the structural advantages of the RSSM architecture for modeling complex multivariate time-series and highlights its significant potential as a robust digital twin for industrial process control.

#### 4.2.2. Analysis of Model Sample Efficiency and Generalization Performance

To rigorously evaluate the model’s generalization capabilities and address the risk of data leakage, the dataset comprising 30,000 discrete timesteps was strictly partitioned in chronological order for all experiments. Random shuffling of data points was explicitly avoided, as it would disrupt the inherent temporal dependencies of the crystallizer’s thermodynamic processes and inadvertently leak future dynamic patterns into the training phase. By employing sequential splitting, the model is trained exclusively on historical trajectories and evaluated on strictly unseen, future operational states. Furthermore, because the data was acquired by tracking randomized temperature setpoints rather than following fixed, repetitive operational cycles, the validation set contains unique transient and steady-state behaviors, ensuring the model is evaluated on its ability to generalize physical dynamics rather than merely recalling periodic patterns.

**Figure 7:**
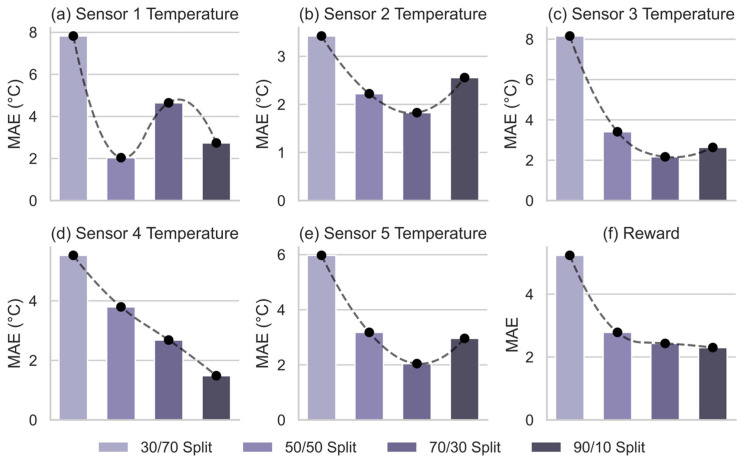
Analysis of Model Performance Dependency on Training Set Splitting Ratios.

This section further quantifies the model’s prediction accuracy as a function of the volume of available training data, providing deeper insights into its sample efficiency and generalization. As shown in Figure 7, the mean absolute error (MAE) on the validation set for the five sensor signals and the reward signal trends downward as the proportion of training data increases across four split ratios (30/70, 50/50, 70/30, and 90/10). For clarity, these ratios follow the format ‘Training %/Validation %’. Specifically, the ‘30/70’ split indicates that 30% of the sequential data was used for training and 70% for validation, whereas the ‘90/10’ split allocates 90% to training and 10% to validation. This trend is expected, as more data enables the model to learn more granular and robust patterns in the system dynamics, thereby constructing a more generalizable internal representation.

Notably, Performance gains are most pronounced when shifting from a 30/70 to a 50/50 data split. Beyond this threshold, additional training data yields diminishing returns as the rate of MAE reduction slows significantly. This suggests that a 50% split provides sufficient information to capture the system’s core dynamics. For high-cost applications such as crystallization, these findings validate the efficiency of the data collection strategy. Switching to a 90/10 split did not universally improve accuracy, as Sensors 2, 3, and 5 showed higher MAE. This decline likely stems from reduced validation diversity, which increases sensitivity to outliers, or potential overfitting, where the model prioritizes memorizing noise over generalizing physical laws. These results demonstrate that maximizing data volume is not always optimal. Instead, a 50/50 or 70/30 split offers the best balance between model robustness and acquisition costs, providing a practical guideline for digital twin deployment in resource-constrained environments.

#### 4.2.3. Validating the Long-Term Reliability of the Model as a Virtual Sensor

**Figure 8:**
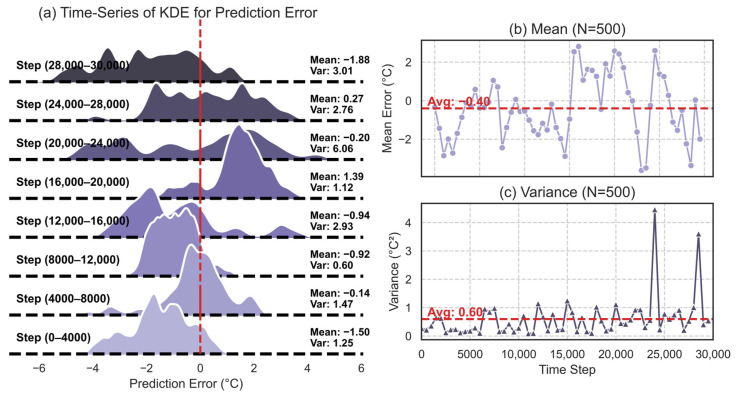
Changes in the Mean and Variance of the Error from the 30,000-Step Autoregressive Prediction.

This experiment analyzes the model’s behavior during an extended 30,000-step autoregressive generation process to evaluate the cumulative effect of prediction errors—a critical metric for generative time-series models. The experiment utilizes a model trained on the previously established 50/50 training-validation split. As illustrated in Figure 8a, an initial seed sequence from the real dataset serves as the starting point. The model predicts the state at t+1; subsequently, this prediction becomes the input for forecasting the state at t+2. This autoregressive process is iterated 30,000 times, guided solely by real-world control feedback without corrections from unobservable environmental temperature data. The resulting 30,000-step prediction-error trajectory is divided into eight consecutive 4000-step windows, which are visualized in the probability density plots in Figure 8a. As shown in the series of Kernel Density Estimate (KDE) plots, the error distribution evolves dynamically. Notably, even in the final window (Steps 28,000–30,000), where cumulative error typically diverges in standard models, the system exhibits stable performance. Given this context, where prediction error does not monotonically accumulate, it strongly suggests that the underlying physical crystallization process exhibits non-stationary dynamics and that the model successfully learns the intrinsic global dynamics of the system.

Furthermore, no significant performance divergence was observed across the 30,000 steps. To better visualize the model’s predictive evolution, the dynamic changes in the mean and variance of prediction errors were calculated over a moving 500-step window. The top panel of Figure 8b (labeled “Mean”) shows that the error mean fluctuates around zero, with its overall average (−0.40) remaining within a narrow and acceptable range. The bottom panel of Figure 8c (labeled “Variance”) indicates that the prediction variance remains generally stable, fluctuating around a mean of 0.60. Although slight fluctuations in variance are observed in the later stages of the trajectory, the error does not exhibit exponential growth, confirming the absence of catastrophic long-term performance degradation. These findings collectively demonstrate the reliability of the RSSM World Model in long-term multi-step autoregression and validate its feasibility as a high-fidelity digital twin and virtual sensor.

**Figure 9:**
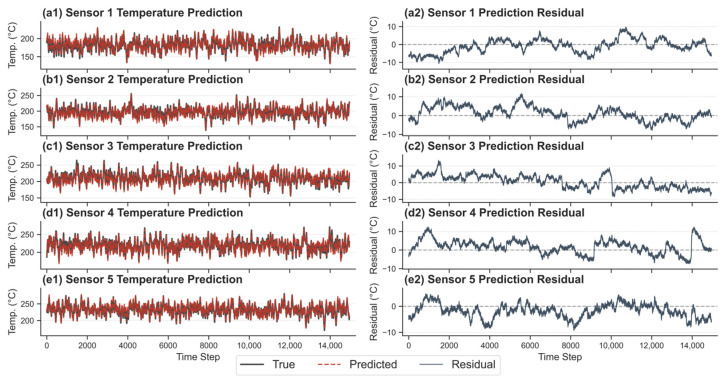
Full-trajectory temperature predictions and residuals for all five sensors on the validation dataset.

To explicitly rule out the hypothesis that the model might simply be learning the long-term statistical average of this unsteady process, a common failure mode that could theoretically cause deceptive performance rebounds when the system naturally reverts to its mean, the full-trajectory predictions and residuals were visualized on the 15,000-step unseen validation set (Figure 9). As explicitly demonstrated in the left panels of Figure 9, the predicted trajectories (red dashed lines) actively and precisely track the high-frequency, non-stationary fluctuations of the true temperatures. A model that defaults to a long-term average would yield flat, smoothed trajectories. Furthermore, the residual plots in the right panels confirm that the prediction errors are tightly constrained around zero, predominantly within a narrow ±10 °C band.

These visual results indicate that the model captures the underlying time-varying thermodynamic dynamics rather than defaulting to a statistical mean. Consequently, the stability observed in Figure 8 is not an artifact of mean reversion. Instead, it reflects the model’s ability to track the physical system as it transitions into a more stable operational phase. This alignment demonstrates that the trained RSSM possesses the necessary robustness to accurately represent complex industrial processes over extended horizons without accumulating errors or “drift toward the mean” often seen in less capable models.

#### 4.2.4. Ablation Study

To systematically evaluate the contribution of the proposed architectural design and the necessity of state information, a series of ablation studies was conducted. The results are summarized in Table 1, comparing the baseline model against the proposed multi-head configuration under different feature settings.

First, the effectiveness of the multi-head structure was examined. As shown in Table 1, the baseline Single-Head (concat. Encoding) model yields a Mean Absolute Error (MAE) of 4.6032 and a Root Mean Square Error (RMSE) of 8.2724. By transitioning to the Multi-Head (concat. Encoding) architecture while maintaining the same feature inputs, the model achieves a substantial performance boost. The MAE decreases by 45.5% to 2.5087, and the RMSE drops by 61.7% to 3.1722. This significant improvement indicates that assigning dedicated prediction heads to distinct sensor segments enables the model to better capture the specific dynamics of each region, overcoming the information bottlenecks inherent in a single-head design. Subsequently, the importance of the ‘absolute temperature’ and ‘temperature difference’ input features was further investigated. The results for the Multi-Head (w/o Abs. Temp.) and Multi-Head (w/o Temp. Diff.) configurations show that removing either feature results in a noticeable performance degradation compared to the whole model. Specifically, the model without absolute temperature has an MAE of 3.5133, while the model without temperature difference has an MAE of 3.0821. In terms of the optimal configuration, the best results are observed in the Multi-Head (concat. Encoding) setup, which integrates both information sources. By concatenating the static system state (absolute temperature) and dynamic trends (temperature difference), the model achieves the lowest error rates across all metrics (MAE 2.5087, Mean Absolute Percentage Error (MAPE) 1.25%). This confirms that the combination of the multi-head architecture with a comprehensive feature set is the optimal strategy for high-fidelity temperature prediction.

### 4.3. RL Model Validity Experiment

This section primarily evaluates the performance of the reinforcement learning (RL) policy, with both training and validation conducted entirely based on the pre-trained RSSM (World Model). The discussion includes comparative experiments on RL training modes, comparisons between traditional control methods and the proposed RL approach, and an analysis of reward function designs. Finally, it details the Bayesian optimization approach used to determine the optimal weighted combination of three sub-rewards: temperature control, smoothness, and control cost.

#### 4.3.1. Training and Performance Validation of the Reinforcement Learning Agent

In the Model-Based Reinforcement Learning (MBRL) framework, the agent learns from a world model trained on data sampled from real-world trajectories, rather than directly interacting with the real environment. Policy optimization is then achieved by generating a large number of simulated trajectories using the learned model. Consequently, the stability and convergence of the training process not only reflect the performance of the RL algorithm itself but also indirectly validate the quality and self-consistency of the underlying world model. In this paradigm, the world model—initialized from states sampled from real trajectories—acts as a highly efficient data augmenter by generating diverse “imagined” experiences. This diversity is critical to prevent the agent from exploiting artifacts or loopholes within the world model, and to ensure the learned policy is effective and generalizable.

**Figure 10:**
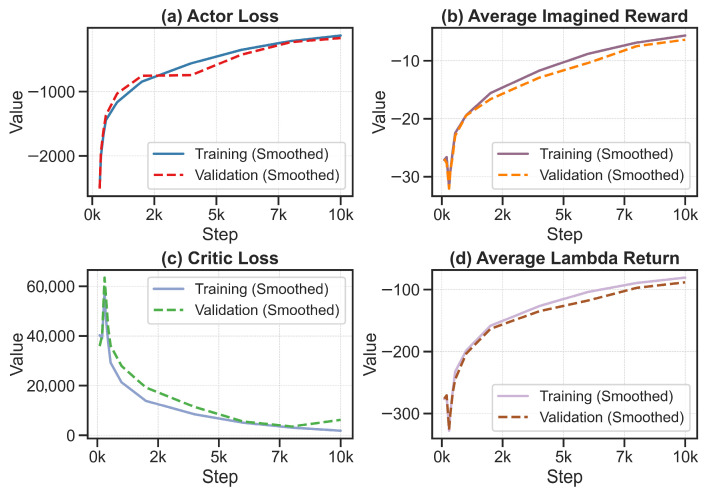
Validation of Stable Convergence and Generalization of the Reinforcement Learning Policy.

The training dynamics of the reinforcement learning policy are illustrated in Figure 10, with the sub-plots presented in sequential order. First, Figure 10a displays the Actor’s loss curve. It shows a steady upward trend, which indicates that the agent is progressively learning to select actions deemed more favorable by the Critic. Next, Figure 10b presents the evolution of the Average Reward. This metric exhibits a clear, stable upward trend before plateauing, demonstrating that the agent is successfully identifying strategies that yield superior immediate outcomes. Subsequently, Figure 10c illustrates the Critic’s loss. The curve rapidly declines from a high initial value before converging smoothly. This indicates that the Critic quickly becomes an accurate estimator of long-term values, providing reliable guidance for the Actor’s updates. Finally, Figure 10d depicts the Episode Return. Similar to the reward curve, it shows a robust convergence to a high steady-state value, confirming that the agent has mastered the task and reached a near-optimal solution within the simulated world.

A compelling feature observed across these plots is the overlap between the training and validation curves. In machine learning, a significant gap typically signals overfitting. Here, the absence of such a discrepancy suggests that the learned policy captures fundamental system dynamics rather than specific ‘imagined’ scenarios, ensuring robust performance in subsequent validation tests.

**Figure 11:**
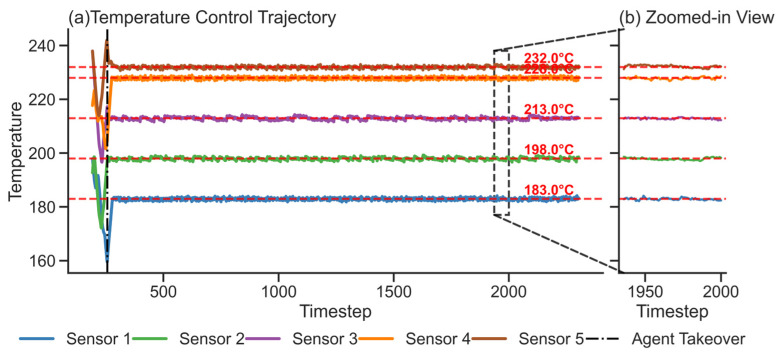
Transient Response and Steady-State Temperature Trajectories under Agent Control.

The performance of the learned reinforcement learning policy is validated in Figure 11, which illustrates an extended long-term control trajectory divisible into two distinct phases. The first phase (Timestep < 64) depicts the initial temperature trajectory before the agent’s intervention, inferred by the RSSM from real-world control data. The second phase begins at Timestep 64, when the agent assumes control and the system initiates a transient response. In stark contrast to the preceding disordered oscillations, this phase is characterized by rapid and goal-directed convergence. All sensor readings rapidly converge towards their respective setpoints. Subsequently, the system achieves a prolonged steady state extending up to 2048 timesteps. To effectively demonstrate the fine-grained local control dynamics within this extensive horizon, the zoomed-in view in Figure 11b details the steady-state behavior over the 1936–2000 timestep interval. Over this entire duration, all temperature trajectories fluctuate tightly around their respective setpoints (red dashed lines), resulting in minimal steady-state error. The negligible amplitude of these fluctuations across the 2048-step horizon compellingly demonstrates the agent’s excellent disturbance-rejection capabilities and robust long-term system stability.

This experiment demonstrates that strategies trained via the MBRL framework exhibit strong generalization capabilities. Not only do they achieve stable convergence during simulation, but they also deliver rapid response, high-precision convergence, and excellent disturbance resilience in practical control applications.

#### 4.3.2. Performance Comparison with Other Control Strategies

The proposed Actor-Critic (AC) agent is compared against three baselines: standard Proportional-Integral-Derivative (PID), fuzzy PID, and Model Predictive Control (MPC). The standard PID is designed to minimize tracking errors using fixed parameters optimized via an extensive grid search of historical data. Building on this, the fuzzy PID accounts for the system’s thermal inertia by dynamically adjusting its parameters online using tailored fuzzy logic rules. To guarantee a rigorous comparison, the MPC baseline is designed to optimize the same reward function and non-linear environment model (RSSM) as the AC agent. It employs a GPU-accelerated Random Shooting method within a standard receding-horizon framework. Crucially, all key MPC hyperparameters, including the prediction and control horizons and candidate sample size, were systematically determined via grid search to ensure optimal baseline performance. Table 2 compares their final performance based on three penalty scores computed from Equation (17), with lower scores indicating better performance. Specifically, the quantitative values presented correspond to the time-averaged penalty terms derived from the reward function. “Control Performance” denotes the Mean Maximum Absolute Error, calculated as the temporal average of the maximum absolute tracking deviations across all five zones. Similarly, “Stability Performance” measures the Mean Maximum Step-wise Fluctuation, reflecting the average peak instantaneous volatility. Finally, “Energy Consumption” represents the Mean Control Action Cost based on the defined actuator usage penalties (Heating: 1.5, Cooling: 1.0, Simultaneous: 2.5, Idle: 0.0); this metric quantifies the algorithmic control effort and actuator usage frequency rather than the total electrical power consumption of the heating elements.

Compared to standard PID controllers, the AC agent significantly improves performance. PID control struggles with the system’s nonlinearity and coupling, often inducing overshoot and unnecessary energy loss due to its lack of explicit cost optimization. Notably, the AC agent also demonstrates substantial advantages over MPC, particularly regarding energy consumption (0.0773 versus 1.1890) within the validated steady-state operating range. Unlike MPC, which relies on computationally intensive online optimization over a finite horizon, the AC agent utilizes offline training to encode long-term strategies. This allows it to execute decisions with global foresight via efficient forward passes, avoiding the short-sightedness often inherent in real-time solvers.

The disparity in energy efficiency further highlights a fundamental difference in optimization capability. To ensure a fair comparison, the MPC baseline employs the same reward weights as the AC agent. However, because the energy penalty weight is relatively small compared to the dominant temperature tracking objective, the MPC solver—constrained by its finite horizon—is driven to greedily minimize tracking error, largely neglecting the marginal energy cost. Crucially, the AC agent’s ability to successfully optimize this subtle energy objective, whereas MPC neglects it, serves as compelling evidence of the proposed strategy’s effectiveness. It demonstrates that the offline-trained policy captures global optima and long-term marginal gains that the finite-horizon MPC overlooks.

#### 4.3.3. Analysis of Reward Function Design

To investigate the critical role of the reward function’s design on the agent’s final performance, an ablation study was conducted focusing on two aggregation operators—mean and max—used to construct the temperature control and stability penalty terms. As defined in Equation (17), the baseline model uses the max operator for both penalties to penalize worst-case performance. Table 3 presents the final evaluation results for four configurations, covering temperature control performance, stability performance, and energy consumption (lower scores indicate better performance).

First, regarding the temperature control penalty, a comparison of configurations using ‘Mean’ as the first component (the first two rows) versus those using ‘Max’ (the last two rows) in Table 3 shows that models employing the max operator achieve significantly superior final temperature control performance. This indicates that in multi-variable, multi-zone temperature control tasks, penalizing the maximum error (max) is more effective than punishing the average error (mean). The mean operator can compensate for poor performance on specific sensors by averaging with good performers, masking significant errors. Conversely, the max operator compels the agent to address the worst-performing sensor—analogous to the “weakest link” principle—ensuring coordinated and precise control across all zones. Second, the analysis of the stability penalty yields a similar conclusion. Comparing configurations that use ‘Mean’ as the second component (rows 1 and 3) versus ‘Max’ (rows 2 and 4), the latter achieve significantly better (lower) final stability scores. This suggests that penalizing the maximum single-step temperature change encourages the agent to generate smoother trajectories. The mean operator can obscure sharp local control actions. In contrast, the max operator effectively suppresses sudden, large-amplitude fluctuations, which are critical for maintaining long-term stability in industrial processes.

In summary, although the (Max, Max) configuration did not achieve the best score in any individual metric, it delivered highly competitive results in both temperature control (0.7155) and stability (0.6137), preventing significant performance degradation across any dimension. This design strikes a near-Pareto-optimal balance, compelling the agent to simultaneously optimize worst-case control accuracy and stability, ultimately learning a control strategy that effectively balances precision and robustness.

#### 4.3.4. Bayesian Optimization

An efficient hyperparameter search strategy was used to systematically determine the optimal weights for each objective function component. Specifically, the modern optimization framework Optuna (version 3.6.1) was utilized, which defaults to the Tree-structured Parzen Estimator (TPE) algorithm—an efficient Bayesian optimization method suited for handling complex non-convex objective functions.

**Figure 12:**
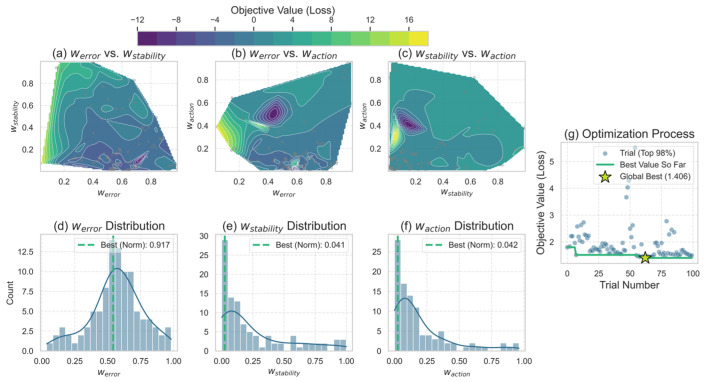
Analysis of Hyperparameter Search Processes Based on Bayesian Optimization.

The optimization objective is to find a set of weights $w_{error}$, $w_{stability}$, and $w_{action}$ as shown in Equation (17). This cost function is the sum of three key performance penalty terms. These measure the deviation between the control outcome and the target, the process volatility, and the energy consumption cost of the control actions. Figure 12 details the optimization process and final results of the 100 trials. The three two-dimensional contour plots in Figure 12a–c illustrate the relationship between any combination of two weight parameters and the final comprehensive cost. Darker colors (deep purple) indicate lower costs and superior performance. Subfigures (a) and (c) clearly suggest that the optimal performance region consistently appears when the $w_{stability}$ value is extremely low (close to 0). This demonstrates that the weight of the stability penalty term significantly impacts the final cost and must be minimized. Consequently, an excessively high $w_{stability}$ would cause the control model to prioritize stability at the expense of the more critical temperature control performance. Additionally, subfigure (b) shows that optimal solutions cluster within the $w_{error}$ range of 0.4 to 0.6, while $w_{action}$ occupies a relatively distinct low-value region. This reveals a delicate trade-off, requiring careful balancing between the error penalty and the penalty for action energy consumption. The loss surface analysis confirms that the key to model performance is assigning a dominant but moderate weight to the error term while imposing smaller penalty weights on stability and energy consumption.

Figure 12d–f presents three histograms of the optimization algorithm’s sampling frequency for each parameter within the search space, with the parameter values corresponding to the global optimum marked in green dashed lines. It is important to note that the annotations in the figures (e.g., “Best (Norm)”) display the normalized values used internally by the optimizer. In contrast, the following discussion refers to the corresponding actual parameter values for physical interpretation. Subfigure (d) exhibits a centralized distribution approximating a normal distribution, with the final optimal actual value determined to be 0.542. This location corresponds to the center of the optimal region identified through the loss surface analysis. The sampling distributions in subfigures (e) and (f) exhibit pronounced right skewness. This indicates that after minimal initial exploration, the optimization algorithm rapidly converged its focus to the low-value region for intensive exploitation, aligning perfectly with observations from the contour plots. The final optimal values found are $w_{stability}\approx 0.024$ and $w_{action}\approx 0.025$.

Finally, Figure 12g illustrates the evolution of the overall cost with the number of trials. During the early optimization phase (approximately the first 25 trials), the algorithm conducted extensive “exploration,” leading to a wide distribution of cost values and significant fluctuations. As trials progressed, the algorithm gradually converged. The global optimum solution (indicated by the star marker) was found around the 60th trial, with a cost value of 1.406. Subsequent trials did not discover a better solution, indicating that the optimization process had successfully converged to a stable optimal region.

A Bayesian optimization strategy implemented with Optuna successfully determined the optimal weight combination. Experimental results indicate that setting weights to, $w_{stability}\approx 0.024$, and $w_{action}\approx 0.025$ achieves optimal performance with a minimum comprehensive cost of 1.406. This visual analysis validates the optimization results and provides deep insights into the contribution of each objective term.

## 5. Conclusions

This study addresses the control challenges associated with complex nonlinear dynamics and partial observability in multi-temperature-zone electric continuous crystallizers by proposing and validating a sensorless closed-loop control framework based on the Recurrent State-Space Model (RSSM). By leveraging historical offline data to construct a high-fidelity industrial digital twin, this study developed a “virtual sensor” capable of real-time inference of latent states. This approach effectively addresses the limitations of deploying physical sensors in extreme industrial environments. It demonstrates the feasibility of using model inference to replace direct measurement for closed-loop control in complex systems.

Experimental results demonstrate that the constructed RSSM exhibits exceptional long-term predictive capabilities, maintaining high stability and accuracy during multi-step autoregressive prediction without significant error accumulation. The Reinforcement Learning (RL) agent trained on this basis significantly outperforms traditional PID and Model Predictive Control (MPC) strategies, specifically improving temperature tracking accuracy by over 67%. Furthermore, through comparative experiments on reward function design for multi-zone cooperative control, this study verified that the Maximum (Max) operator holds greater optimization potential than the Mean operator when handling multi-variable coupled deviations. By penalizing the worst-performing temperature zone, the Max operator effectively prevents local severe errors from being masked by overall averaging, thereby ensuring consistent precision and stability across all control regions.

In summary, this research not only provides a novel paradigm for intelligent control in sensor-constrained environments but also demonstrates the immense potential of offline Model-Based Reinforcement Learning (MBRL) in enhancing industrial automation.

## 6. Discussion

This work validates a sensorless closed-loop control framework based on historical offline data, effectively mitigating challenges arising from model uncertainty and partial observability in complex industrial processes, such as multi-temperature-zone continuous crystallizers. The core value of this research lies in validating the engineering feasibility of the “virtual sensor” paradigm. By deploying the RSSM to infer unobservable system states in real time, this work provides a viable technical pathway for achieving intelligent control under sensor-limited or harsh operating conditions, thereby reducing reliance on invasive physical hardware.

A critical insight from this study is the distinct performance advantage of the proposed Reinforcement Learning (RL) agent over the widely adopted Model Predictive Control (MPC). This superiority is attributed to their differing optimization paradigms. MPC relies on online finite-horizon optimization, which often leads to myopic decisions that prioritize immediate tracking errors over marginal energy costs. In contrast, the RL agent performs global policy optimization during the offline training phase. This process encodes long-term control strategies directly into the neural network, allowing the agent to execute complex, long-horizon decision-making through a single forward pass. This approach not only avoids the computational latency of iterative online solvers but also optimizes global value, resulting in substantial improvements in energy efficiency.

Despite these achievements, limitations inherent to offline learning and the current experimental scope must be acknowledged. The framework’s performance boundary is fundamentally determined by the initial offline dataset, which was primarily collected under steady-state conditions. Consequently, the model lacks exposure to extreme out-of-distribution (OOD) states or dynamic variations such as equipment aging. However, field investigations indicate that industrial crystallizers are already maintained within a suitable thermal envelope by empirical baseline controls (e.g., fixed-power heating). Therefore, the proposed MBRL framework is positioned not as a cold-start controller for system recovery, but rather as an intelligent “fine-tuner” that synergizes with existing methods. It optimizes efficiency within a safe operational space by dynamically adjusting temperatures based on virtual sensing. Beyond data constraints, a secondary limitation lies in the standard RSSM architecture, which may be less computationally efficient than emerging sequence modeling techniques when handling extremely long temporal dependencies.

Future research will focus on the following directions: (1) Exploring the integration of linear complexity sequence models (such as structured state space models, SSMs, e.g., Mamba) into RSSMs to replace their recurrent kernel functions, thereby enhancing modeling efficiency and long sequence processing capabilities. (2) Developing hybrid learning protocols enabling agents pre-trained on offline data to undergo constrained online fine-tuning, thereby adapting to process dynamics that evolve or deviate from the original dataset distribution. (3) Designing conservative and safety-constrained control models to guarantee that the system operates strictly within a defined safe operational space. By embedding explicit safety boundaries to prevent control actions from driving the system into extreme out-of-distribution (OOD) states, this approach ensures the validity of both prediction and control models, simultaneously eliminating the need to sample all theoretical states during data collection exhaustively. (4) Extending existing perceptual networks to construct high-fidelity multimodal datasets incorporating richer physical field information. By introducing multi-source heterogeneous data, this addresses the issue of insufficient information in steady-state data, thereby enabling the training of prediction and control models with higher robustness. (5) Conducting rigorous experimental validation to bridge the “sim-to-real” gap. This involves assessing the accuracy of RSSM-inferred states relative to physical sensor measurements and evaluating the RL agent’s performance under actual industrial operating conditions to confirm the practical applicability of the proposed framework.

Ultimately, this study presents a robust solution for complex industrial process control, offering methodological insights for integrating offline reinforcement learning with intelligent manufacturing systems.

## Figures and Tables

**Table 1:** Evaluation of the multi-head architecture and state encoding methods.

Model Configuration	MAE (°C)	RMSE (°C)	MAPE (%)
Single-Head (concat. Encoding)	4.6032	8.2724	3.2139
Multi-Head (w/o Abs. Temp.)	3.5133	5.2220	2.2023
Multi-Head (w/o Temp. Diff.)	3.0821	5.3510	2.1998
Multi-Head (concat. Encoding)	2.5087	3.1722	1.2507

**Table 2:** Performance Comparison of Different Control Strategies.

Method	Control Performance	Stability Performance	Energy Consumption
PID	2.7362	8.2724	1.3230
Fuzzy PID	2.2208	2.2375	1.3148
MPC	1.9908	1.7091	1.1890
Actor-Critic	0.7155	0.6137	0.0773

**Table 3:** Performance Comparison of Different Reward Function Designs.

Configuration (Temp., Stab.)	Control Performance	Stability Performance	Energy Consumption
Mean, Mean	1.0029	0.7012	0.0695
Mean, Max	0.9432	0.5912	0.0801
Max, Mean	0.7019	0.8918	0.0724
Max, Max	0.7155	0.6137	0.0773

## Data Availability

The data supporting this study’s findings are available from the corresponding author upon reasonable request.

## References

[1] Skogestad S. (2003). Simple analytic rules for model reduction and PID controller tuning. J. Process Control.

[2] Zhao X., Sun Y., Li Y., Jia N., Xu J. (2024). Applications of machine learning in real-time control systems: A review. Meas. Sci. Technol..

[3] Garriga J.L., Soroush M. (2010). Model predictive control tuning methods: A review. Ind. Eng. Chem. Res..

[4] Hou Z.-S., Wang Z. (2013). From model-based control to data-driven control: Survey, classification and perspective. Inf. Sci..

[5] Qin S.J., Chiang L.H. (2019). Advances and opportunities in machine learning for process data analytics. Comput. Chem. Eng..

[6] Tseng M.-L., Tran T.P.T., Ha H.M., Bui T.-D., Lim M.K. (2021). Sustainable industrial and operation engineering trends and challenges Toward Industry 4.0: A data driven analysis. J. Ind. Prod. Eng..

[7] Kiran B.R., Sobh I., Talpaert V., Mannion P., Al Sallab A.A., Yogamani S., Pérez P. (2021). Deep reinforcement learning for autonomous driving: A survey. IEEE Trans. Intell. Transp. Syst..

[8] Levine S., Kumar A., Tucker G., Fu J. (2020). Offline reinforcement learning: Tutorial, review, and perspectives on open problems. arXiv.

[9] Rajasekhar N., Radhakrishnan T., Samsudeen N. (2025). Exploring reinforcement learning in process control: A comprehensive survey. Int. J. Syst. Sci..

[10] Seborg D.E., Edgar T.F., Mellichamp D.A., Doyle F.J. (2016). Process Dynamics and Control.

[11] Kadlec P., Gabrys B., Strandt S. (2009). Data-driven soft sensors in the process industry. Comput. Chem. Eng..

[12] Gao Z., Li Z., Wang J., Luo H., Shi X., Chen M., Li Y., Zuo L., Du Z., Xiao Z. (2023). Funasr: A fundamental end-to-end speech recognition toolkit. arXiv.

[13] Higgins K., Nyssen O.P., Southern J., Laponogov I., Veselkov D., Gisbert J.P., Kanonnikoff T.F., Veselkov K. (2025). The Helicobacter pylori AI-clinician harnesses artificial intelligence to personalise H. pylori treatment recommendations. Nat. Commun..

[14] Qian L., Zhou W., Wang Y., Peng X., Yi H., Zhao Y., Huang J., Xie Q., Nie J.-Y. (2025). Fino1: On the Transferability of Reasoning-Enhanced LLMs and Reinforcement Learning to Finance. arXiv.

[15] Dulac-Arnold G., Levine N., Mankowitz D.J., Li J., Paduraru C., Gowal S., Hester T. (2021). Challenges of real-world reinforcement learning: Definitions, benchmarks and analysis. Mach. Learn..

[16] Garcıa J., Fernández F. (2015). A comprehensive survey on safe reinforcement learning. J. Mach. Learn. Res..

[17] Fujimoto S., Meger D., Precup D. Off-policy deep reinforcement learning without exploration. Proceedings of the International Conference on Machine Learning.

[18] Agarwal R., Schuurmans D., Norouzi M. An optimistic perspective on offline reinforcement learning. Proceedings of the International Conference on Machine Learning.

[19] Kumar A., Zhou A., Tucker G., Levine S. (2020). Conservative q-learning for offline reinforcement learning. Adv. Neural Inf. Process. Syst..

[20] Kaelbling L.P., Littman M.L., Cassandra A.R. (1998). Planning and acting in partially observable stochastic domains. Artif. Intell..

[21] Yu T., Kumar A., Chebotar Y., Hausman K., Levine S., Finn C. (2021). Conservative data sharing for multi-task offline reinforcement learning. Adv. Neural Inf. Process. Syst..

[22] Moerland T.M., Broekens J., Plaat A., Jonker C.M. (2023). Model-based reinforcement learning: A survey. Found. Trends Mach. Learn..

[23] Matsuo Y., LeCun Y., Sahani M., Precup D., Silver D., Sugiyama M., Uchibe E., Morimoto J. (2022). Deep learning, reinforcement learning, and world models. Neural Netw..

[24] Grieves M., Vickers J. (2016). Digital twin: Mitigating unpredictable, undesirable emergent behavior in complex systems. Transdisciplinary Perspectives on Complex Systems: New Findings and Approaches.

[25] Tao F., Zhang M. (2017). Digital twin shop-floor: A new shop-floor paradigm towards smart manufacturing. IEEE Access.

[26] Mnih V., Badia A.P., Mirza M., Graves A., Lillicrap T., Harley T., Silver D., Kavukcuoglu K. Asynchronous methods for deep reinforcement learning. Proceedings of the International Conference on Machine Learning.

[27] Hafner D., Lillicrap T., Fischer I., Villegas R., Ha D., Lee H., Davidson J. Learning latent dynamics for planning from pixels. Proceedings of the International Conference on Machine Learning.

[28] Smith J.T., Warrington A., Linderman S.W. (2022). Simplified state space layers for sequence modeling. arXiv.

[29] Gu A., Dao T. Mamba: Linear-time sequence modeling with selective state spaces. Proceedings of the First Conference on Language Modeling.

[30] Garnett R. (2023). Bayesian Optimization.

[31] Akiba T., Sano S., Yanase T., Ohta T., Koyama M. Optuna: A next-generation hyperparameter optimization framework. Proceedings of the 25th ACM SIGKDD International Conference on Knowledge Discovery & Data Mining.

[32] Bergstra J., Bardenet R., Bengio Y., Kégl B. (2011). Algorithms for hyper-parameter optimization. Adv. Neural Inf. Process. Syst..

[33] Xu B., Kang Y., Zhao X., Yan H., Ju F. (2025). Partially observable Markov decision process framework for operating condition optimization using real-time degradation signals. J. Qual. Technol..

[34] Sun H., Hu Y., Luo J., Guo Q., Zhao J.J.B. (2025). Enhancing HVAC control systems using a steady soft actor–critic deep reinforcement learning approach. Buildings.

[35] Khodarahmi M., Maihami V. (2023). A review on Kalman filter models. Arch. Comput. Methods Eng..

